# Perspectives of disabled adults on healthcare professionals role in promoting physical activity in China A reflexive thematic analysis

**DOI:** 10.1038/s41598-025-97921-4

**Published:** 2025-05-08

**Authors:** Wei Wang, Jie Xiang, Yang Huo, Husam Alfaifi, Stacey Pope, Brett Smith

**Affiliations:** 1 Department of Sport and Exercise Sciences, Durham University, Durham, UK; 2 Department of Rehabilitation, The Affiliated Hospital of Xuzhou Medical University, Xuzhou, Jiangsu China; 3 The Second School of Clinical Medicine, Xuzhou Medical University, Xuzhou, Jiangsu China; 4 Ezhou Central Hospital, Ezhou, Hubei China

**Keywords:** Disabled persons, Physical activity, Healthcare personnel, Rehabilitation, Qualitative research, Rehabilitation, Health care

## Abstract

Healthcare professionals (HCPs) have been identified as key messengers for disabled adults in promoting physical activity (PA). However, few studies revealed the disabled adults’ views and preferences for HCPs in PA. This study aimed to understand disabled adults’ perceptions and experiences of the role of HCPs in promoting PA in China. Forty-one Chinese disabled adults participated in one-to-one semi-structured interviews, recruited by three purposive sampling strategies: maximum variation, criterion-based and snowball sampling. Data from interviews were analysed using reflexive thematic analysis. Three themes were identified: (1) mastery beyond the medical, (2) title clarification within a HCP hierarchy, and (3) balancing medical and lay words with accessibility. Participants valued the role of HCPs in integrating PA within medical care but noted gaps such as insufficient guidance on transitioning PA to daily life, lack of diverse options, and limited support for fostering autonomy in PA quality participation. Misunderstandings about HCP roles hindered effective healthcare interactions, while clear, disability-sensitive communication was essential for building trust and fostering PA participation. This study highlights the need for HCP training to improve personalised PA guidance, role clarity, and communication skills alongside policy changes to better support disabled adults in PA participation. Additionally, future research should involve a broader sample to improve these PA strategies and assess their impact.

**Supplementary Information:**

The online version contains supplementary material available at 10.1038/s41598-025-97921-4.

## Introduction

Physical activity (PA) participation is a critical component of public health, essential for preventing non-communicable diseases, and improving the health and well-being of disabled people^1–4^. However, many disabled individuals remain largely inactive^2,5^, which can lead to a lower quality of life for disabled people and increasing economic costs for society^2^. Disabled adults face numerous specific challenges in achieving effective PA participation, which further reduces the quality and quantity of their PA participation^6–11^. Traditional PA promotion strategies for disabled people focus on who communicates and how to communicate PA information and knowledge^2^. In this context, PA messengers play a vital role in delivering information about how to be physically active.

Healthcare professionals (HCPs), including doctors in general practices, physiotherapists, occupational therapists, and nurses in the context of rehabilitation, have been identified as crucial messengers in PA promotion^2,12–14^. These HCPs are regarded as trustworthy messengers by disabled people to deliver PA information, valuing their ability to offer personalised advice, support, and motivation for PA participation^12^. Despite this trust, the methods by which HCPs can effectively provide PA information and communicate with disabled adults remain unclear^15^. The lack of effective ways for HCPs to communicate and deliver PA information can impact the success of PA interventions. For example, inadequate communication that fails to address the patients’ unique needs has been shown to weaken the therapeutic relationship with HCPs, and reduce the effectiveness of HCPs in the medical context^16^.

Moreover, there is limited research specifically examining the views and preferences of disabled people for HCPs in PA, particularly in the context of China. Much evidence, based on the perceptions of HCPs, indicates the challenges they face in promoting PA to the targeted population. These challenges include a lack of PA-related knowledge, skill, and time^15,17^. However, these studies mainly highlight HCPs’ perceptions and views overlooking the audiences’ demands. Although a few studies highlight the resources required by disabled people, along with the factors impacting HCPs from the perspectives of disabled people, these studies mainly concentrate on PA participation rather than the perceived role of HCPs from the perspective of disabled people^18,19^. Furthermore, this evidence is mainly based on Western context, which may not fully capture the unique cultural, social and healthcare systems in China^18,19^. It is vital that different national cultural contexts are examined so differences and similarities across the globe can be identified and to support global policies like the World Health Organisation global action plan on PA^20^.

Thus, it is important to understand the preferences and perceptions of disabled adults concerning HCPs and PA promotion, which is essential for developing effective strategies to overcome barriers and challenges to PA participation, particularly in the context of China. To address this gap, this study aims to explore disabled adults’ perceptions and preferences for HCPs in PA promotion within the context of China, in order to know how to better support PA participation among disabled adults. The findings of this study could guide research and practical work involving HCPs and stakeholders, providing recommendations for more effective interventions by HCPs to promote PA among disabled adults.

## Methods

### Philosophical foundation

This study is underpinned by an interpretive paradigm, where reality is dynamic, multifaceted, and shaped by individuals’ subjective interpretations of their experiences and environments, and knowledge is co-constructed through social interactions and interpretation^21,22^. Adopting an interpretive paradigm allowed us to explore the roles of HCPs in PA promotion among disabled adults in China, focusing on participant-centred insights and contextual understanding. This philosophical foundation guided the research design, ensuring alignment with the study’s objectives and methodological choices. It enabled an in-depth exploration of disabled adults’ perceptions of HCPs, reflecting their lived experiences and cultural contexts.

### Study design

A qualitative approach was conducted from June 2022 to February 2023 to recruit disabled adults to discuss their views about PA and HCPs. The presentation of this paper follows the standards for reporting qualitative research (SRQR)^23^.

### Participants and recruitment

The primary criteria for this study were that participants needed to be: (a) aged 18 years or over; (b) with a disability/disabilities; and (c) living in China. Eligibility was assessed through initial screening interviews conducted by the first author to ensure participants met the inclusion criteria. All types of disabilities were included and self-reported. Three purposive sampling strategies, maximum variation, criterion-based and snowball sampling, were employed to ensure a larger diversity of voices from participants^24^. The maximum variation strategy considered different disabilities, ages, sex, geographical locations (living places), education, marital status and household incomes based on the previous literature^25–27^. Three different settings were also captured: rehabilitation settings, villages in rural areas and city centres in China due to the differing accessibility to HCPs and PA resources in these settings^28,29^. Snowball sampling was utilised to recruit participants with a disability who might provide additional data and boost variation plus informational power. Participants were asked to recommend other participants who might have PA experiences different from what they had provided.

Participants were recruited by the authors in collaboration with disability organisations, community leaders, healthcare institutions and peers with disabilities. Organisers and representatives from these groups assisted in contacting potential participants or providing their contact information with consent. Initial contact was made via telephone or WeChat, after which the authors verified eligibility and invited participants to join the interviews. Rather than adopting data saturation as this is incompatible with a reflective thematic analysis^30^, recruitment continued until information power^31^ was achieved. Information power refers to the process of identifying the size of a sample for a qualitative study that uses analyses like a reflexive thematic analysis^31^. Following recommendations^31^, to determine sufficient information power we considered when recruiting and through the ongoing analysis (a) the aim of the study, (b) sample specificity, (c) use of established theory or framework, (d) quality of dialogue, and (e) analysis strategy. For example, this study had a broad clear aim, the sample specificity was dense by focusing on disabled adults in China, an established framework was applied within the use of PAMF, the interview dialogues were semi-structured, and the analysis strategy was the case^31^. To add further rigour, two phases of participant recruitment were conducted to ensure higher information power. The first phase was conducted between June and October 2022. However, there were no participants with mental health issues and only one person with a hearing impairment after the first phase of recruitment. To address these gaps, the second phase was conducted from November 2022 to February 2023, focusing on recruiting more participants with hearing impairments and/or mental health conditions. By adopting this two-phase recruitment, we ensured the representation of underrepresented groups, enabling richer and more comprehensive insights into participants’ experiences and preferences on HCPs in PA promotion.

The study received ethical approval from the Ethical Committee of the Department of Sport and Exercise Sciences, Durham University (Application reference: SPORT-2020‐12‐11T17_36_43‐xzcj41). All procedures were conducted in accordance with Durham University’s ethical policies on research involving human participants and adhered to these relevant guidelines and regulations. All participants provided informed consent before the interviews. A small voucher (e.g., one Renminbi) was employed as a modest means of appreciation for participants’ time and effort. To ensure that the voucher did not bias their responses, participants were explicitly informed that their participation was voluntary, and their responses would remain confidential, with no consequences linked to the voucher. Thirty pounds was provided for the professionals as gatekeepers and sign language translators who worked in organisations or hospitals to support recruitment and data collection. The funds were provided by the Department of Sport and Exercise Sciences, Durham Student Union, and the St Chad’s College at Durham University, without any conflict of interest.

A total of 41 participants were interviewed; Table 1 includes demographic information. Supplemental materials include a copy of the interview guide. Participants (*n* = 10) in the rehabilitation setting reported that they had physical disabilities. In the community, 31 participants reported a range of impairment experiences (for example, physical, visual, hearing, attention deficit hyperactivity disorder, autism). Most participants were from Eastern China and mainly resided in Jiangsu Province, Guangzhou, Beijing, and Shanghai. All interviews were recorded, and participants were assigned anonymised codes: XR for participants from rehabilitation settings and CC for those from community settings, followed by a unique identifying number.

**Table 1:** Demographic information of participants in the interview (*N* = 41).

Characteristics		*n*	%	Characteristics		*n*	%
Age (year)	18–34	19	46.3	Setting	Community	32	78.0
	35–59	18	43.9		Rehabilitation	10	24.4
	60 and above	4	9.8	The household income	The low-income group	11	26.8
Sex	Male	25	61.0		The middle-income group	10	24.4
	Female	16	39.0		The high-income group	18	43.9
Marital Status	Unmarried	19	46.3		Prefer not to say	2	4.9
	Married	16	39.0	The educational Level	Lower or primary school	8	19.5
	Widowed	1	2.4		Middle school	10	24.4
	Divorced	4	9.8		High school and equal qualification education	2	4.9
	Prefer not to say	1	2.4		College and above	21	51.2
Impairments	Physical	25	61.0	Living areas	Urban	27	65.9
	Physical with Neurological diverse conditions	11	26.8		Rural	14	34.2
	Visual	9	22.0	Occupation	Unemployed	8	19.5
	Hearing	5	12.2		Student	4	9.8
	ADHD	1	2.4		Have a job	29	70.7
	Autism	1	2.4	Religion	No religious belief	40	97.6
					Have a religious belief	1	2.4

n = number of participants; % = n/all participants (*N* = 41) * 100.

### Data collection

Data were collected through semi-structured online interviews and in-person interviews due to the individual demands of interviewees, and geographic and political issues. Each interview used an interview guide to support conversations (see Supplemental materials). The guide was informed by the Physical Activity Messaging Framework (PAMF), drawing on its core principles and components, including understanding targeted audiences and the context of the messages, message content and delivery^32^. These core principles and components of the PAMF were considered to ensure that the questions comprehensively addressed participants’ perceptions and experiences of HCPs in promoting PA, enhancing the relevance and depth of the data collected.

The interview guide was piloted before the main study with co-authors and two peers with disabilities who shared similar characteristics with the target participants. These individuals were not included in the final sample. Pilot testing was conducted by the first author, an experienced healthcare professional with training and expertise in qualitative research and the field of PA, to refine the guide and ensure its relevance to the study objectives. When necessary (i.e. participants with hearing impairments), interviews were supported by a professional sign language translator. To ensure linguistic accuracy, the professional translator was briefed by the first author on the study’s aims and the importance of maintaining neutrality and accuracy when conveying both questions and responses. The translator repeated the content in both sign language and spoken language, facilitating clear communication between the researcher and participants with hearing impairments. For people with mental disabilities, at their request, carers or family members supported them in the interview. Interviews lasted between 18 and 194 minutes, with an average duration of approximately one hour per person, depending on the participants’ level of engagement and responses.

### Data analysis

**Fig. 1:**
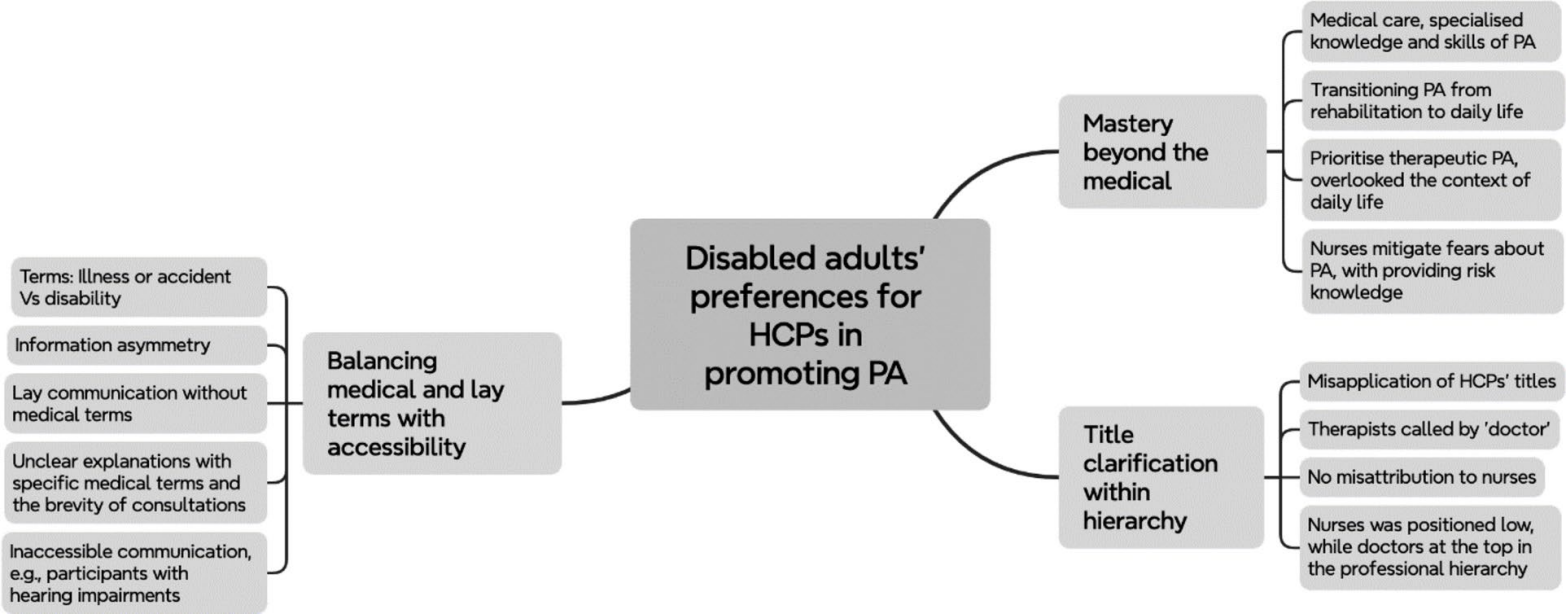
Final thematic map for disabled adults’ preferences regarding HCPs in promoting PA analysis.

A reflexive thematic analysis was used to analyse the data, comprising six phases: (1) familiarisation, (2) coding, (3) initial theme generation, (4) theme development and review, (5) theme refining, defining and naming, and (6) writing up^33,34^. This process of reflexive thematic analysis enabled a multifaceted exploration of data, encompassing deductive, inductive, critical and reflexive perspectives at any time^33,34^. The deductive perspectives drew on the PAMF^32^, using its constructs to guide the analysis related to HCPs’ roles in promoting PA for disabled adults. This said, we did not ‘push’ the framework into the data. Analysis also included examining the data inductively to understand what else might be of value and what gaps there might be. We were constantly open to new ideas, thought critically about the PAMF, and considered how the PAMF may at times not align with data, if applicable. Microsoft Excel for Mac served as a tool to manage the data, facilitating both the organisation of the data and the visualisation of relationships between codes, sub-themes, and superordinate themes. Initial data were analysed in Chinese allowing for a more nuanced and culturally sensitive examination of the material. Following the creation of the results, a translation occurred from Chinese to English. This linguistic shift was pivotal, enabling comprehensive discussions with critical peers and the eventual presentation of findings. Final themes are presented in Fig. 1.

### Reflexivity

Reflexivity was important to ensure the rigour and trustworthiness of this study^26,28^, which was embedded throughout the research process, involving continuous introspection, intersubjective reflection, and collaborative peer engagement to enhance credibility and contextual relevance^35^. This approach allowed us to navigate the complex cultural and experiential dimensions inherent in the study. The first author, a non-disabled Chinese physiotherapist with academic training in both China and the UK, brought a unique combination of cultural sensitivity and professional expertise to the research. While this background provided valuable insights, it also introduced potential biases. These biases were addressed through reflective practices and intersubjective critique by the research team^35^, which included co-authors with diverse cultural and professional backgrounds in qualitative research. This diversity within the team fostered discussions that challenged interpretations. Collaborative reflection also played a central role in the analytical process^35^. Co-authors and peers here then acted as “critical friends” engaging in discussions that enriched the depth and nuance of the analysis^36^. For example, critical friends questioned the relevance of interview questions (e.g., why and how to ask these questions, etc.), the embedded position in the study (e.g., strangers, friends, patients, etc.), and the implicit meanings of terms or themes (e.g., mastery, hierarchy, etc.) in the interpretation, prompting deeper reflexive engagement with the data. These interactions enhanced reflexivity by encouraging the first author to be critical of her assumptions, interpretative patterns and potential biases throughout the research process. Insights shared by interview participants further informed and refined interpretations, ensuring that the findings reflected participants’ lived experiences. Peer debriefings were conducted throughout the research process to refine data collection, analysis, and the presentation of results, strengthening the study’s credibility and trustworthiness. This reflexive and collaborative approach ensured that the study’s findings were not only credible but also contextually sensitive, providing a nuanced understanding of the roles of HCPs in promoting PA among disabled adults.

## Results and discussion

Three themes were identified: (1) mastery beyond the medical, (2) title clarification with hierarchy, and (3) balancing medical and lay terms with accessibility. These themes, generated from participants’ responses to interview questions about HCPs and their roles in promoting PA, highlight the interaction between the PAMF principles and participants’ lived experiences, while uncovering insights beyond the framework’s scope but important to understanding the roles of HCPs in PA promotion for disabled adults.

### Theme 1: mastery beyond the medical

Disabled adults underscored the essential role of HCPs in providing medical care and personalised PA guidance, offering supervision and advice to support PA in the context of rehabilitation. This finding emphasises PAMF’s principles of message content and delivery. Participants who underwent rehabilitation emphasised the specialised knowledge and skills of doctors and therapists in providing good healthcare services that were focused on improving physical functions and medical conditions. As one participant said:

These doctors and therapists are all experts, which puts me at ease when performing any movement. They teach me various simple exercises to increase muscle strength and improve joint mobility. They closely monitor me as I exercise in the therapy room and promptly correct any mistakes. The movements they teach are straightforward and easy to learn. Their supervision ensures that we stay focused and do not slack off during our exercises. (XR09)

Additionally, participants emphasised that HCPs played an important role in helping disabled adults in transitioning PA from within rehabilitation to daily life. HCPs facilitated adaptive activities, defined as tailored PA designed to accommodate the specific needs of disabled individuals, such as wheelchair mobility training and balance improvement exercises, which aim to enhance disabled adults’ psychological health, confidence and independence in daily life for disabled adults. As one participant shared:

For me, this is a very important course in the Stylistics Therapy Department (Chinese:文体疗法科) at the rehabilitation centre. They taught me how to use a wheelchair in various situations, such as overcoming obstacles, going up and down slopes, and turning the wheelchair. This has allowed me to move around and start to dance again. (CC06)

However, doctors and physiotherapists tended to prioritise therapeutic PA centred on the medical condition and its clinical treatment^37,38^. Disabled adults felt that HCPs overlooked the importance of integrating PA into their daily lives, which is crucial for helping them transition from rehabilitation to daily activities. Some participants reported that this therapeutic PA was commonly standardised, repetitive and monotonous. This familiarity and repetition of therapeutic PA, which led to a feeling of mastery among disabled adults themselves, did not fully address disabled adults’ desire to engage in diverse and autonomy-enhancing PA. A sole focus on this therapeutic PA also diminished the credibility of HCPs from the viewpoint of disabled adults. HCPs were not deemed good sources of PA information as the professionals prescribed simply a repetitive therapeutic approach to PA. In line with one previous study^16^, participants were moreover reluctant to participate in this repetitive PA with HCPs due to financial issues. This gap underscores the limitations in PA message content and delivery from HCPs for disabled adults. As one participant stated:

The actions taught by the doctors (therapists) are quickly learned if you stay here a bit longer. I have been here for four years, and the new doctors (therapists) may not know as much as I do. Everyone’s treatment is more or less the same; there is no significant difference. (XR08)

I don’t want to go to the hospital often now, going to the hospital costs money, and now for me, spending money is useless. (XR10)

Additionally, nurses were noted for their crucial roles in mitigating the feeling of PA-related risk among disabled adults. Many participants expressed a fear of engaging in PA due to the potential risks and accidents, such as falls and injuries. Nurses played a crucial role in alleviating these fears by monitoring fall-related incidents and providing timely support for disabled adults within the context of rehabilitation. They worked alongside doctors and therapists, ensuring a safe and supportive environment for rehabilitation. Previous studies have highlighted the multifaceted roles of nurses in rehabilitation settings, which include assessment, coordination of care, and providing emotional support to patients^39^. By addressing both the physical and emotional needs of disabled adults, nurses help foster a more secure and encouraging atmosphere for engaging in PA. As participants said:

Nurses frequently inquire about our condition and caution us to exercise carefully. In contrast, doctors and therapists typically supervise our activities directly, offering immediate correction and guidance on our movements. (XR06)

These aforementioned findings highlight different roles and preferences of nurses in PA among disabled adults compared to those of doctors and therapists, which are in line with one recent study which suggested that the roles and preferences of nurses and therapists in PA were slightly different^40^. The study pointed out that nurses primarily focus on the patients’ rest and safety, whereas therapists are more concerned with the patients’ PA, emphasising various therapeutic activities to help patients recover and maintain physical functions^40^. This difference in roles and tendencies advocates a complementary dynamic in the rehabilitation process but can also present coordination challenges in PA promotion.

This theme highlights the need to expand PA delivery beyond therapeutic settings, incorporating diverse, autonomy-enhancing activities that balance safety, engagement, and practicality. While this theme aligns with PAMF’s principles of content and delivery, it also underscores the limitations of standardised therapeutic approaches and the importance of differences in the roles of HCPs in sustaining PA participation for disabled adults.

### Theme 2: title clarification within a HCP hierarchy

Participants frequently expressed confusion about the titles of HCPs they interacted with, raising dilemmas in their efforts to become physically active. Participants highlighted key groups of HCPs: physiotherapists, occupational therapists, doctors and nurses. The word *doctor* was often incorrectly extended beyond medical doctors to include physiotherapists and occupational therapists, especially among older disabled adults living in rural areas. Participants explained using the term *doctor* for HCPs was a way to honour them when they could not distinguish their specific roles. As participants stated:

The best thing about being in a hospital is that doctors and nurses are around when doing exercises. I’m not sure about therapists who they are; I just call them ‘doctor’ or ‘teacher so-and-so’. (XR02)

It’d be awkward to call them (*HCPs*) anything else. Calling someone by their full name directly seems rude. (CC04)

As suggested, this lack of awareness about the distinct roles of physiotherapists and occupational therapists influenced their trust towards HCPs and the credibility with which they were perceived to promote PA^41,42^. The uncertain and often incorrect title usage influenced participants’ perceptions^43^, leading to misunderstandings, biases in healthcare interaction and tensions in therapeutic relationships between HCPs and patients^16,43–46^. This uncertainty and misapplication resulted in ineffective PA promotion by HCPs for disabled adults. Clear identification of what each HCP is about and does is crucial to minimise this misapplication and enable disabled people to make informed healthcare decisions^43^. These findings emphasise the importance of clarity in HCPs’ titles for disabled adults’ PA participation, which could be useful in building positive therapeutic relationships between HCPs and disabled adults.

However, the uncertainty and misattribution of titles did not extend to nurses. Participants explained that nurses were mostly women and wore distinct uniforms that helped distinguish their identification from other HCPs. As participants stated:

If a nurse is an expert, I will listen to their advice on PA. But if I were to compare, I would give precedence to the doctor’s advice. (XR06)

Nurses are easy to know. They had hats and wore different clothes from others. (CC04)

This perceived hierarchy among HCPs, which places nurses in a subordinate role compared to doctors and therapists, aligns with previous studies in the context of rehabilitation^47,48^. This subordinate perception of nurses could negatively impact the efficacy of rehabilitation and PA promotion efforts by nurses among disabled adults.

This theme aligns with the PAMF’s focus on messenger identity, highlighting how the roles and credibility of HCPs shape the effectiveness of PA promotion among disabled adults. Misattributions and unclear role identification undermine the perceived credibility of HCPs, hindering their capacity to deliver impactful PA messages. While not directly aligned with the PAMF’s predefined components, these findings offer critical insights into how role ambiguity and hierarchical perceptions influence trust in HCPs. Addressing these challenges requires efforts to enhance awareness of HCP identities and contributions to improve PA promotion and build positive therapeutic relationships among disabled adults. Furthermore, challenging medical hierarchies remains an important task in PA promotion.

### Theme 3: balancing medical and Lay terms with accessibility

Disabled adults held different preferences for disability-related terms when communicating with HCPs that impacted on their PA participation. Participants had a preference for terms, such as *illness or accident* over *disability* when discussing PA with HCPs within rehabilitation contexts. This preference reflected a reluctance to accept their disability and an optimism towards recovery, especially during the early stage of rehabilitation or the transitional period from medical care to societal settings. The use of these medical terms underscored the influences of medical models of disability on PA promotion within healthcare contexts. As one participant stated:

I am a patient. Since I got the illness, I’ve been consistently exercising. Keeping doing exercise, I may recover completely one day. There may be a miracle. (XR09)

In contrast, some participants, who had not experienced rehabilitation, resisted being labelled a *patient* from HCPs, suggesting a preference for the social model of disability over the medical model of disability. Scullion critiqued these medicalised labels by HCPs, such as *patients*, arguing that they negatively impacted the self-identity of disabled individuals during the transitional phases by reinforcing a weakened self-perception in line with the medical model of disability^49^. In the words of one participant:

You go to the hospital when you are sick or seriously ill. I am disabled, not ill. I am not a patient to do exercise. So I do not frequently prefer HCPs in PA participation. (CC23)

These different preferences for disability-related terms indicate varying attitudes towards disability among disabled adults, reflecting a divergence in self-perception and identity during different disability phases. These varying attitudes towards disability among disabled adults underscore the necessity for HCPs to use appropriate disability-related terms in promoting PA for disabled adults, tailored to the different phases of disability they experienced. As participants stated:

I come back here every year for rehabilitation after my illness. It’s been three or four years now. I am a patient not like normal persons. I take the initiative to exercise more here. Those healthcare workers call me *uncle*; it motivates me to stay here. (XR09)

I became disabled because of an accident when I was very young. It’s been many years now, and I only see a doctor when I’m sick. I’m not a patient, and I don’t want to go to the hospital. If I were to consult about exercise, I wouldn’t want to be treated as a patient or be told I have an illness. Being referred to in a non-medical way encourages me to stay active. (CC04)

Participants also emphasised the need for lay language in communicating about PA with HCPs in rehabilitation contexts. Effective lay communication was suggested to foster positive relationships between HCPs and disabled adults, creating an inclusive environment in rehabilitation settings. This inclusive environment was highly valued by disabled adults as it enhanced their sense of belonging and trust with HCPs in rehabilitation settings, influencing disabled adults’ decision to participate in PA and extend their stay in rehabilitation settings. As participants explained:

Doctors and therapists work very seriously and have a good attitude. I can ask them anything I don’t understand when I am doing exercise in the rehabilitation department. They have the patience to answer my questions and listen to me during my exercises. I do exercise every day except for Sunday. There is no discrimination here. Everyone around is similar. In the beginning, I couldn’t accept the reality of my disability. I met so many people in the same situation in the hospital. I realised that I’m not the only one like this in the world. At home, there is no such environment without discrimination. Sometimes we train together and have chats. We feel like family together. Actually, the hospital environment had a significant impact on me. (CC06)

The doctors are busy and don’t have enough time to answer questions. Sometimes we don’t understand the professional terms the doctors use, sometimes they are in English. We haven’t been educated, we don’t understand. In the therapy room, after getting to know the therapists, sometimes we ask questions while they are treating other patients, but we definitely can’t do that every day. (XR09)

These quotes highlight the importance of effective lay language in building trust and positive relationships between HCPs and disabled adults for promoting PA. One previous study found that therapeutic relationships were often tense due to inadequate communication and a lack of effective conflict mediation in public hospitals in China^16^. The absence of effective lay language, coupled with the complexity of medical terms and brief consultation with unclear explanations from HCPs, can exacerbate information asymmetry and misunderstandings among disabled adults, reducing the effectiveness of PA promotion they suggested. Therefore, it is important for HCPs to use lay languages and create inclusive environments to support disabled adults in PA participation in China.

Regarding the inclusive environment for promoting PA, accessibility is an important factor for effective communication between HCPs and disabled adults. Participants with hearing impairments particularly emphasised the difficulties they faced in accessing communication with HCPs in PA participation, such as the need for sign language assistance. This inaccessibility in communicating with HCPs is compounded by logistical challenges, such as online registration and the high cost of medical treatment and services. As participants stated:

Going to the hospital is quite inconvenient; you have to register, and now many registrations have to be done online or on a machine by yourself. There might be nurses to help with registration, but they are very busy; sometimes one person has to help many people. For us with hearing impairments, going to the hospital is actually more troublesome, the doctors also can’t understand our sign language, and we can’t speak out. (CC28)

Registration costs money. If possible, we still hope to have some free consultations with doctors in the community to help us out. (XR09)

This theme reflected PAMF’s focus on message framing and inclusive communication, which also revealed the deeper impact of terminology on identity and trust. Factors, such as information asymmetry, economic issues, and the dominance of the medical model of disability, influence the effectiveness of communication between HCPs and disabled adults. These findings revealed limitations in PAMF’s practical application, addressing systematic barriers such as financial issues. Communication barriers, such as the use of complex medical terms and brief consultations, coupled with logistical challenges like online registration and high treatment costs, further exacerbate these issues. To address these challenges, more accessible, community-based healthcare consultations should be developed to reduce financial barriers and be tailored to the specific needs of disabled adults. These solutions could include subsidised healthcare services, and financial assistance programs to offer low-cost or free consultations. By addressing these barriers and promoting effective lay communication, HCPs can better support PA participation among disabled adults, fostering a more inclusive and accessible environment for disabled adults to promote PA.

### Strengths and limitations

This study is the first qualitative investigation into disabled adults’ preferences for how HCPs promote PA within the context of China. A key strength of this research is its novelty. By focusing on disabled adults in China, this study offers original evidence to support PA promotion in healthcare settings. The insights gathered from participants shed light on their perceptions and experiences with HCPs, highlighting the potential role of these professionals in promoting PA. Additionally, these insights suggest potential ways to strengthen therapeutic relationships between healthcare professionals and patients in China.

The authors acknowledge several limitations of the study. The sample, consisting of a convenience group of disabled adults, was predominantly from Eastern mainland China. The recruitment period and online interview methods restricted the sample size, particularly among adults with mental illnesses. This limitation may have resulted in a selection bias, with more motivated individuals sharing their experiences. Future research should aim for a more diverse sample, including a broader range of disabled adults and geographic areas, particularly those with mental illnesses, to provide a more comprehensive insight.

## Conclusion

The study provides critical insights into the preferences of disabled adults for how HCPs effectively promote PA in China. These findings highlight the importance of HCPs extending expertise beyond the medical roles to incorporate various supportive directions, clarifying professional titles to enhance therapeutic relationships, applying appropriate medical terms during transitional periods, and improving lay language to foster an inclusive and accessible environment in rehabilitation settings. To address these gaps, HCP training programs should include targeted knowledge and skills that prioritise personalised PA guidance, title clarity and inclusive communication approaches. Additionally, policy initiatives should focus on embedding such training programs into healthcare systems, ensuring that PA promotion becomes accessible, sustainable, and effective for disabled adults. Recognising that HCPs cannot and should not be the sole messengers of PA, it is also vital to train HCPs in collaborative practices. This includes working with other professionals and community stakeholders to create a unified approach to PA promotion. Interprofessional collaboration is essential for future policy development, enabling coordinated efforts across sectors to deliver comprehensive and impactful strategies. The authors recommend that HCPs, rehabilitation departments and other stakeholders collaborate with disabled adults to develop and co-produce PA resources that effectively promote PA participation^50^. This collaborative approach will ensure the effectiveness of resources, which are personalised to meet the unique needs and preferences of disabled adults, thereby enhancing their engagement in PA.

## Electronic supplementary material

Below is the link to the electronic supplementary material.

Supplementary Material 1

Supplementary Material 2

## Data Availability

Data is provided within the manuscript or supplementary information files. Individual participant data in the study will not be made available publicly. For further detailed data access policy and procedure, please contact wei.wang@durham.ac.uk.
