# Supplementary material for: Perspectives of disabled adults on healthcare professionals role in promoting physical activity in China A reflexive thematic analysis
Source: Sci Rep. 2025 May 8;15:16040. doi: 10.1038/s41598-025-97921-4 (PMC12062288; doi:10.1038/s41598-025-97921-4)
Supplement: Supplementary file 1 — Supplementary Material 1 [file 41598_2025_97921_MOESM1_ESM.docx]

# **Supplemental Materials**

## *Appendix 1: The interview guide for data collection*

Part 1: Introduction （Not Recorded Part）

(To introduce myself ) My name is Wei Wang, a PhD student at Department of sport and exercise sciences, Durham University. I am studying communication for disabled people to be more physically active.

(Thank participants and explain the purpose of the study) Thank you very much for participating in this interview. This study aims to explore communication resources by your experiences and understanding of disability and physical activity to promote physical activity. Thus, your any answers and questions are important for us.

(To explain the interview process) I will ask you several questions about disability and physical activity messaging that are no right or wrong answers. You are encouraged to share your experiences, opinions and perceptions of disability and physical activity messaging. You are free to answer or reject these questions without limited time and content. Please, to be honest when you are answering questions. If you do not want to answer it, please let me know. You are voluntary and free to miss out on any question or stop taking part at any time without giving a reason.

An important thing that I would like to tell you is that this interview will be recorded to help the researcher who can gain any useful and vital information. Please rest assured, everything you say will remain completely confidential, and the information will only be used by the Research Team. Any published research that might result from such discussions will not contain your name or identifying information.

The interview will last for approximately 30 mins to 1 hour. During this time, I would like to explore a number of questions on this topic. If you have any questions about the disability and physical activity messaging, I will be more than happy to answer these at the end of the session. Do you have any questions about the interview?

If you do not have any questions, I would like to turn on audio recorders and start the interview now.

Part 2: Interview content (Recorded Part)

| Questions |
| --- |
| Section 1 Personal details  (May I first ask you personal information? If there is not convenient to say the content you can tell me do not want to say, these are casual, you rest assured that the content we are also absolutely confidential.) |
| 1. May I have your name or nickname ；  2. How old are you now ;  3. Gender/Sex ;  4. The type of disability and when  5. Occupation:  6. Religion:  7. Marital status:  8. Living place:  9. Household income:  10. Education level: |
| Section 2 Disability  （Next we would like to know about your disability, and we may talk about your views on disability.） |
| 1. Would you like to tell us about your experience or stories with the disability?   ( e.g., what/ when / how did it happen/ how it influences your life ) |
| 2. What do you think about the current situation of people with disabilities around you in terms of your daily life. |
| Section 3 Physical activity |
| 1. What do you think about physical activity?  (if necessary, explain the definition of physical activity) |
| 2. What does physical activity mean to you?  (e.g., to your health, emotion, working, studying, and relationship with others) |
| 3. Can you share your experiences of doing physical activity?  (e.g., what types, how long and frequency, what is the feeling when you were doing it. ) |
| 4. What kinds of physical activities do you prefer or not prefer to do?  (Based on the survey findings, give the top 3 options and last 3 options separately as examples, ask what do you think about these physical activities ?) |
| 5. What are the reasons that you do physical activity?  (Based on the survey findings, give the top 3 options and last 3 options, ask what do you think about these?) |
| 6. What could motivate you to do physical activity? |
| 7. What are the barriers or challenges for you (disabled adults) to do physical activity in your experience?  (Give examples if needed: Individual factors, social factors, environmental factors or other progressive factors) |
| 8. Where would you like to do physical activity? (Give examples and ask why) |
| 9. What do you think about physical activity in China (or in your community or the hospital) according to your own experience? |
| Section 4 Physical activity messaging  （explanation of physical activity messaging if necessary ） |
| 1. What do you think about physical activity messaging in China and in your community? |
| 2. How do you gain physical activity knowledge generally?  (ask the reason, and give examples from the survey findings, ask what do they think about these ways)? |
| 3. What is the best messaging format for you?  (Based on the survey findings, give the top 3 options and last 3 options, ask what do you think about these?) |
| 4. What is the best information or knowledge (give an example if needed) of physical activity for you to be more physically active?  (ask the reason, and based on the survey findings, give the top 3 options and last 3 options, ask what do you think about these?) |
| 5. What are the barriers or challenges for you to gain education about physical activity ? |
| 6. Who are the best providers or messengers for you to gain physical activity messages ?  (Why them / why not others, and based on the survey findings, give the top 3 options and last 3 options, ask what do you think about these? ) |
| 7. Where is the best place for the message to be delivered for you?  (ask the reason, and based on the survey findings, give the top 3 options and last 3 options, ask what do you think about these?) |
| 8. How often and how long will be message be delivered to you? And at what time is suitable for you?  (ask the reason, why not the other exmaples from the suvey findings) |
| 9. What do you think about an app to give you some physical activity advice based on your particular situation? |

Is there anything else you would like to add about getting and staying physically active?

This is my contact approach if you would like to keep in touch and talk to someone about any problems or difficult feelings about physical activity.

## *Appendix Two: Data analysis*

In the first phase of familiarisation, I actively immersed myself in the data through a three-fold process: 1) active listening, reading and re-reading, 2) critical engagement, and 3) familiarising notes. Adopting active listening supported me in developing an understanding of the primary points addressed in each interview. Manual transcription was conducted after the active-listen playback, and interesting sections were highlighted in Microsoft Word. When the transcription of all interviews was completed, I extensively reviewed the transcripts, reading them many times and transferring them to an Excel sheet for systematic organisation. The practice of taking familiarising notes commenced during the interview, which is also a part of my diary, reflecting the journey of my PhD study. These notes included the initial data trends, potentially interesting points, and my thoughts and feelings regarding both data and the analytical process (see Figure 1). Adopting these processes has helped me to identify appropriate information related to research questions, though it is quite time-consuming and requires a degree of patience.

| 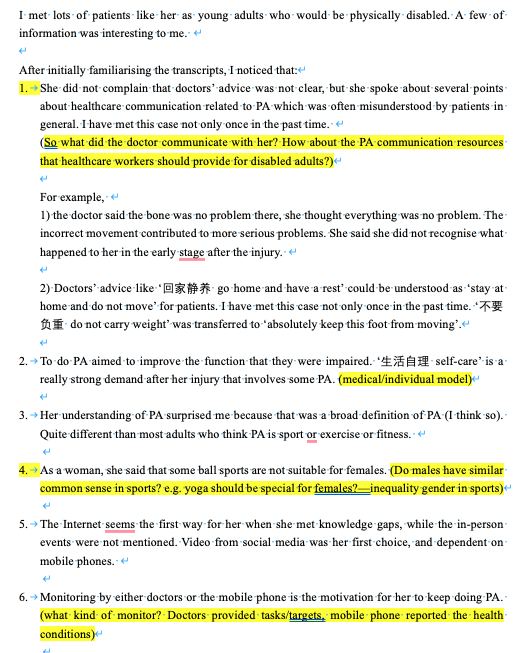 |
| --- |

Figure 1 Examples of preliminary notes during the phase of familiarisation

During the coding phase, I employed a three-tiered coding approach to generate concise and meaningful labels that align with my research questions (Byrne, 2022). The first level of coding centred on research questions. All information that might be useful in addressing my research questions was coded. I systematically worked through the entire dataset and tried to attend to each item with equal consideration. The second level of coding addressed potential theoretical frameworks derived from data, which were partly stimulated by the literature reviewed and relevant theoretical assumptions. The third level of coding was influenced by both the results of the questionnaire and considerations for preparing the initial themes. This iterative coding process allowed me to deepen my familiarity with the data, refining and discarding identity codes as needed to better interpret themes.

All iterations of coding were conducted using Microsoft Excel for Mac, providing a structured framework for review. This allowed me to document codes in the columns, facilitating the tracking of code evolution in relation to each respective text. Tracking the evolution of codes and presenting it in the Excel spreadsheet afforded me signposts and waypoints to which codes may return should a particular approach to coding prove unfruitful. The Excel spreadsheet served as a dynamic tool, highlighting specific transcript sections assigned to each code. Crucially, this approach streamlined the application of different codes to the same transcript sections, offering flexibility in the coding process.

Figure 2 presents a brief example of the preliminary coding process of one interviewee’s transcript, capturing a tracking code change. The columns in the figure delineate the coded number of interviews (Column one), the data item provided by the interviewee (Column two), the initial codes (Column three), and the fourth iteration of data items (Column four). In line with RTA, these codes are brief and are effectively able to stand on their own, offering sufficient detail and informing the underlying commonality with other items in relation to my research questions. Supervisors and peers acted as critical friends to discuss the coding and themes in order to achieve richer interpretations of meaning.


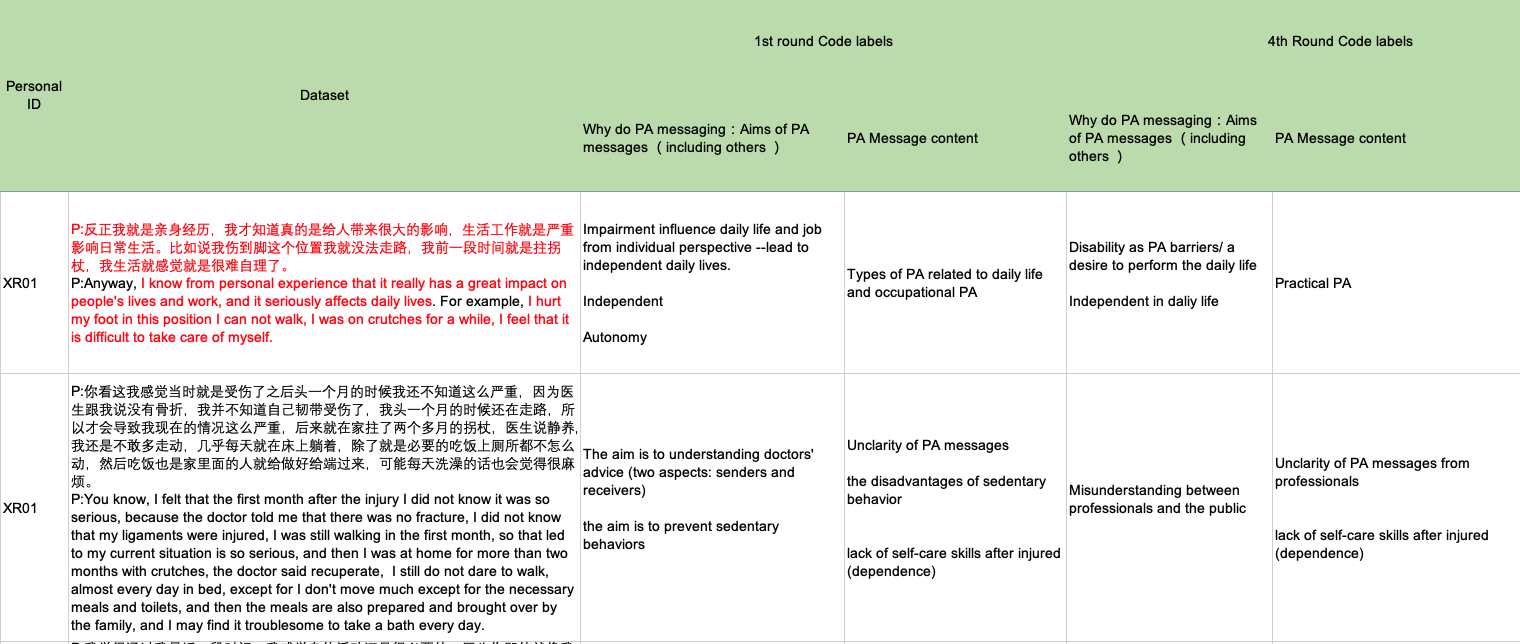


Figure 2 Examples of the preliminary coding and a tracking code change

For the phase involving the initial theme generation, I assembled codes to generate initial themes when I completed the first cycle of coding all data items with regard to my research questions. The codes of data items then underwent a thorough review and analysis to actively construe relationships among the different codes that share a common underlying concept or feature of data. This process led to the development of a sub-theme or a theme. These constructed codes and themes were characterised by their distinctiveness yet interconnected nature, contributing to the creation of a cohesive and clear representation of the dataset. Thus, at the end of the phase, I produced a thematic map that collates codes and data items relative to their respective themes. In cases where certain codes did not align with the overall analysis, they were classified under a miscellaneous theme. This miscellaneous category served as a holding space, allowing for further consideration in subsequent phases or potential removal from the analysis.

During the phase of theme development and review, I recursively reviewed the candidate themes carefully, considering their alignment with the coded data items and the overarching dataset. In order to enhance the candidate themes within meaningful interpretations of the data and address the research questions, two levels of review were conducted. The first-level review focused on the relationship among the data items and codes informing each theme and sub-theme that makes a logical argument and contributes to the overall interpretation and narrative of the data. Moving to the second level, the emphasis shifted to assessing the themes in relation to the entire dataset, as a way of providing the most appropriate interpretation of the data in relation to the research questions, rather than descriptive topic summaries. The final thematic framework, which emerged from these two-level reviews, ensured that each identified theme coherently contributed to the overarching narratives.

In the fifth phase, refining, defining and naming themes produced a detailed analysis of the thematic framework. To refine and define themes, I selected data items to serve as extracts with the aim of providing an analytical description. This involved an interrogation of the important interpretation about what participants said, and contextualising this interpretation in terms of the existing literature. In the process of naming themes, I tried to craft the final names of themes to be concise, informative and memorable, which stand as the titles of Chapters Four to Seven, reflecting the essence of the analysis and guiding readers toward the core findings.

The task of Phase six was to write up the report of themes which not only occurred at the end of the analysis but were, rather, interwoven into the entire process of RTA. Moreover, I established the order of themes which was connected in a logical and meaningful manner by considering the research questions. This ensures that the narrative unfolds in a way that is both cohesive and aligned with the overarching objectives of the study.

## *Appendix 3: All Participants’ Demographic Information*

| **No** | **Code** | **Length of recording** | **Sex** | **Age**  **(year)** | **Types of impairment** | **Education** | **Marital status** | **Household income (RMB)** | **Occupation** | **Living area** |
| --- | --- | --- | --- | --- | --- | --- | --- | --- | --- | --- |
| 1 | XR 01 | 23mins48s | F | 27 | Physical | College | Unmarried | Less than 100,000 | Waitress | Rural |
| 2 | XR 02 | 26mins23s | F | 60 | Physical/Neural | High school | Married | Around 150,000 | Retirement | Urban |
| 3 | XR 03 | 18mins10s | F | 55 | Physical | Middle school | Married | Not reported | Freelance | Urban |
| 4 | XR 04 | 19mins33s | F | 53 | Physical (spinal injury) | Did not finished middle school | Married | Around 50,000 | worker | Rural |
| 5 | XR 05 | 19mins53s | M | 56 | Physical (spinal injury) | Middle school | Married | More than 100,000 | worker | Urban |
| 6 | XR 06 | 24mins59s | M | 59 | Physical (spinal injury) | Primary school | Married | Around 50,000 | Wood workers | Rural |
| 7 | XR07 | 1hour04mins05s | M | 61 | Physical/Neural | Primary school | Married | No income | Unemployed | Rural |
| 8 | XR08 | 1hour11mins21s | M | 44 | Physical/Neural | Middle school | Divorced | Low income | Unemployed | Urban |
| 9 | XR09 | 1hour31mins01s | M | 51 | Physical/Neural | College | Divorced | 7-80,000 | Retired | Rural |
| 10 | XR10 | 19mins53s | M | 59 | Physical/Neural | Primary school dropout | Widowed | No income | Unemployed | Urban |
| 11 | CC01 | 37mins57s | F | 25 | Vision | Bachelor | Unmarried | No income | Student | Urban |
| 12 | CC02 | 42mins34s | F | 39 | Vision | Did not finish primary school | Married | Less than 5,000 | Unemployed | Urban |
| 13 | CC03 | 1hour16mins39s | M | 30 | Vision | Middle school | Unmarried | Less than5,000 | Massager | Rural |
| 14 | CC04 | 1hour30mins16s | M | 42 | Physical | Bachelor | Prefer not to say | More than 100,000 | Freelance | Urban |
| 15 | CC05 | hearing impairment, word chatting | F | 24 | Hearing | High school | Unmarried | 10,000-50,000 | worker | Urban |
| 16 | CC06 | 3hours14mins | M | 37 | Physical | Bachelor | Married | More than 100,000 | Wheelchair dancer | Urban |
| 17 | CC07 | 1hour15mins53s | M | 32 | Vision | College | Unmarried | 10,000-50,000 | Ping pang athlete | Urban |
| 18 | CC08 | 1hour42mins43s | M | 23 | Vision | College | Unmarried | Low income | Student | Urban |
| 19 | CC09 | 1hour09mins22s | M | 30 | Vision | PhD | Unmarried | More than 10,000 | Post doc | Urban |
| 20 | CC10 | 35mins26s | F | 42 | Physical | Middle school | Married | NA | Unemployed | Rural |
| 21 | CC11 | 1 hour 52 mins 57s | F | 22 | Vision | Middle school | Unmarried | Less than 10,000 | Athlete/Swimming | Rural |
| 22 | CC12 | 59mins19s | M | 21 | Vision | Middle School | Unmarried | 50,000-100,000 | Small business | Rural |
| 23 | CC13 | 59mins42s | M | 34 | Physical | Middle school | Unmarried | 12000 | Unemployed | Urban |
| 24 | CC14 | 1hour1mins | F | 32 | Physical | Bachelor | Unmarried | NA | Company staff | Urban |
| 25 | CC15 | 56mins34s | F | 26 | Physical | Bachelor | Unmarried | Less than 10,000 | Unemployed | Urban |
| 26 | CC16 | 1h47mins13s | M | 40 | Physical | Bachelor | Divorced | Around 10,000 | Small business | Urban |
| 27 | CC17 | 1h40mins04s | M | 30 | Physical | Bachelor | Unmarried | Around 20,000 | Company staff | Urban |
| 28 | CC18 | 1hour13mins54s | M | 27 | Physical | Bachelor | Unmarried | 50,000 | Company staff | Rural |
| 29 | CC19 | 1hour25mins50s | M | 31 | Physical | College | Unmarried | 7-80,000 | Company staff | Urban |
| 30 | CC20 | 1hours12mins13s | M | 42 | Physical | College | Married | less than 20,000 | Own business/wheelchair | Urban |
| 31 | CC21 | 46mins58s | M | 32 | Vision | Bachelor | Unmarried | 10,000 | Massager | Urban |
| 32 | CC22 | 1hour43mins41s | M | 47 | Physical | Bachelor | Devoiced but has girlfriends | 60,000 | Unemployment/retired/wheelchair | Urban |
| 33 | CC23 | 1hour04mins30s | F | 61 | Physical | Illiteracy | Married | Less than 5,000 | Farmer/unemployed | Rural |
| 34 | CC24 | 42mins09s | M | 42 | Physical/Neural | Primary school | Married | 50,000 | Farmer/unemployed | Rural |
| 35 | CC25 | 58mins57s | F | 39 | Physical/Neural | Middle school | Married | 20,000 | Retirement of disease | Rural |
| 36 | CC26 | 19mins14s | M | 53 | Physical/Neural | Primary school | Married | 1-50,000 | Farmer/unemployed | Rural |
| 37 | CC27 | video with signal language translator：1hour24mins23s | F | 44 | Hearing | College | Married | 100,000 | worker | Urban |
| 38 | CC28 | video with signal language translator：1hour24mins24s | M | 26 | Hearing | Bachelor | Unmarried/LGBT | 100,000 | apple company staff | Urban |
| 39 | CC29 | video with signal language translator：1hour30mins | F | 60 | Hearing | College | Married | 100,000 | retired | Urban |
| 40 | CC30 | 40mins42s | F | 21 | ADHD | Bachelor | Unmarried | >1000,000 | student | Urban |
| 41 | CC31 | 40mins45s | M | 23 | CP | College | Unmarried | NA | unemployed | Urban |

XR: from rehabilitation settings; CC: from community settings.

## *Appendix 4: Standards for Reporting Qualitative Research (SRQR)*^1^

| **Topic** | **Item** | **Yes/No** | **Page(s)** |
| --- | --- | --- | --- |
| **Title and Abstract** | | | |
| Title | Concise description of the nature and topic of the study Identifying the study as qualitative or indicating the approach (e.g., ethnography, grounded theory) or data collection methods (e.g., interview, focus group) is recommended. | Yes | Main document: 2 |
| Abstract | Summary of key elements of the study using the abstract format of the intended publication; typically includes background, purpose, methods, results, and conclusions. | Yes | Main document: 2 |
| **Introduction** | | | |
| Problem formulation | Description and significance of the problem/phenomenon studied; review of relevant theory and empirical work; problem statement. | Yes | Main document: 4-5 |
| Purpose or research question | Purpose of the study and specific objectives or questions. | Yes | Main document: 5 |
| **Methods** | | | |
| Qualitative approach and research paradigm | Qualitative approach (e.g., ethnography, grounded theory, case study, phenomenology, narrative research) and guiding theory if appropriate; identifying the research paradigm (e.g., postpositivist, constructivist/ interpretivist) is also recommended; | Yes | Main document: 5-6 |
| Researcher characteristics and reflectivity | Researchers’ characteristics that may influence the research, including personal attributes, qualifications/experience, relationship with participants, assumptions, and/or presuppositions; potential or actual interaction between researchers’ characteristics and the research questions, approach, methods, results, and/or transferability. | Yes | Supplemental material: 1 and 6-10 |
| Context | Setting/site and salient contextual factors. | Yes | Main document: 5-9 |
| Sampling strategy | How and why research participants, documents, or events were selected; criteria for deciding when no further sampling was necessary (e.g., sampling saturation). | Yes | Main document: 6 |
| Ethical issues pertaining to human subjects | Documentation of approval by an appropriate ethics review board and participant consent, or explanation for lack thereof; other confidentiality and data security issues. | Yes | Main document: 6 |
| Data collection methods | Types of data collected; details of data collection procedures including (as appropriate) start and stop dates of data collection and analysis, iterative process, triangulation of sources/methods, and modification of procedures in response to evolving study findings. | Yes | Main document: 8-9 |
| Data collection instruments and technologies | Description of instruments (e.g., interview guides, questionnaires) and devices (e.g., audio recorders) used for data collection; if/how the instrument(s) changed over the course of the study. | Yes | Main document: 8-9 |
| Unites of study | Number and relevant characteristics of participants, documents, or events included in the study; level of participation (could be reported in results). | Yes | Main document:7  Supplemental material: 12-14 |
| Data processing | Methods for processing data prior to and during analysis, including transcription, data entry, data management and security, verification of data integrity, data coding, and anonymization/deidentification of excerpts. | Yes | Main document: 8-9  Supplemental material: 7-11 |
| Data analysis | Process by which inferences, themes, etc., were identified and developed, including the researchers involved in data analysis; usually references a specific paradigm or approach. | Yes | Main document: 9  Supplemental material:7-11 |
| Techniques to enhance trustworthiness | Techniques to enhance trustworthiness and credibility of data analysis (e.g., member checking, audit trail, triangulation). | Yes | Main document:9  Supplemental material:7-11 |
| **Results/findings** | |  |  |
| Synthesis and interpretation | Main findings (e.g., interpretations, inferences, and themes); might include development of a theory or model, or integration with prior research or theory. | Yes | Main document: 10-19 |
| Links to empirical data | Evidence (e.g., quotes, field notes, text excerpts, photographs) to substantiate analytic findings. | Yes | Main document: 10-19 |
| **Discussion** | |  |  |
| Integration with prior work, implications, transferability, and contribution(s) to the field | Short summary of main findings; explanation of how findings and conclusions connect to, support, elaborate on, or challenge conclusions of earlier scholarship; discussion of scope of application/ generalizability; identification of unique contribution(s) to scholarship in a discipline or field. | Yes | Main document: 10-19 |
| Limitations | Trustworthiness and limitations of findings | Yes | Main document: 19-20 |
| Conflicts of interest | Potential sources of influence or perceived influence on study conduct and conclusions; how these were managed. | NA  (No conflicts of interest) | Main document: 7 |
| Funding | Sources of funding and other support; role of funders in data collection, interpretation, and reporting. | Yes | Main document: 6-7 |

1. O’Brien, B. C., Harris, I. B., Beckman, T. J., Reed, D. A. & Cook, D. A. Standards for reporting qualitative research: a synthesis of recommendations. *Acad. Med. J. Assoc. Am. Med. Coll.* **89**, 1245–1251 (2014).
